# The specific of development tasks in old age

**DOI:** 10.1192/j.eurpsy.2022.1672

**Published:** 2022-09-01

**Authors:** A. Konovalova, K. Maslova

**Affiliations:** 1 I.M. Sechenov First Moscow State Medical University, Department Of Pedagogy And Medical Psychology, Moscow, Russian Federation; 2 Lomonosov Moscow State University, Faculty Of Psychology, Moscow, Russian Federation

**Keywords:** elderly age, development tasks, development in old age, integrity, wisdom

## Abstract

**Introduction:**

The presentation considers the peculiarities of late adulthood, different views on the periodization of older ages (World health organization, I. Burnside, H.S. Pryazhnikov).

**Objectives:**

The research is aimed at studying the peculiarities of late adulthood.

**Methods:**

The method of work is a bibliographic analysis.

**Results:**

Reveals the structure of psychological age (concept by L.S.Vygotsky), the specificity of the development in late adulthood and features of the social situation of development. Reveals modern ideas of ageing as a process not only of involution and loss, but also a process of continued development. The greatest attention is paid to the peculiarities of development tasks at older ages and the difficulties faced by older people trying to cope with them. There are the brief overview of the positions of C.G.Jung, A.Adler, E.Erikson, R.Peck, G.M.Bryugman, A.G.Liders, N.S.Pryazhnikov, E.E.Sapogova, I.V.Shapovalenko, V.I.Slobodchikov, G.A.Zuckerman, etc. regarding the development tasks in late adulthood. The comparison of the development tasks of early and late age periods by G.M. Bryugman, which shows that the tasks of aging worse defined, at least sequentially ordered, and the results of solution of development tasks is less predictable than in earlier ages.

**Conclusions:**

We can say that in old age is important not only the task of adjusting to different changes of pace of life, quality of life, social circle, etc., and overcome the negative aspects of aging but also issues of self-development. As the primary development task in late adulthood is considered an achievement of his own integrity and finding the meaning of life.

**Disclosure:**

No significant relationships.

